# Supplementary material for: Molecular sorting of nitrogenase catalytic cofactors
Source: bioRxiv. 2025 Jan 21:2025.01.21.634024. Preprint. [Version 1] doi: 10.1101/2025.01.21.634024 (PMC11785038; doi:10.1101/2025.01.21.634024)
Supplement: Supplement 1 [file media-1.pdf]

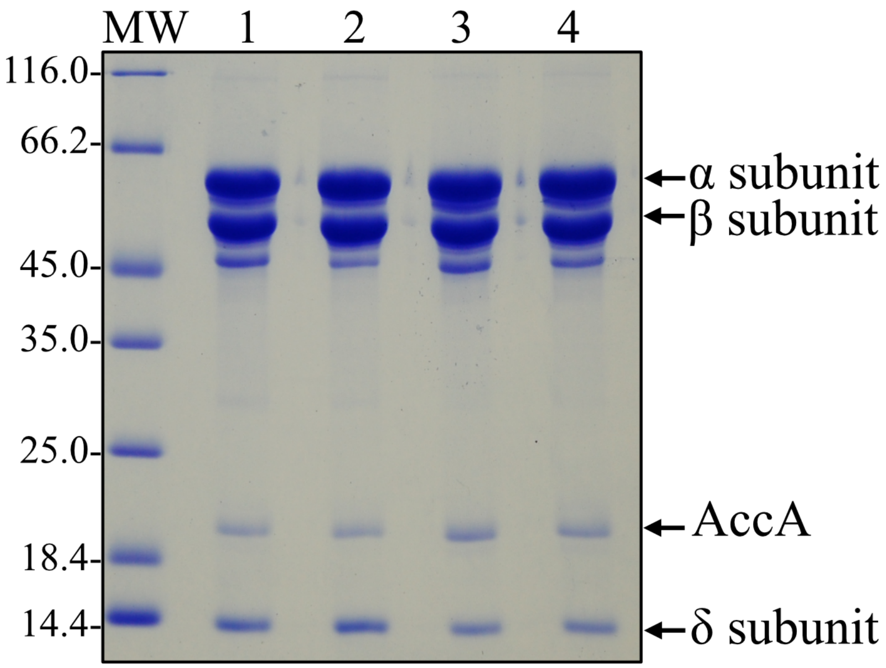

Figure S1. Coomassie blue stained SDS-PAGE of FeFe proteins purified from different strains and growth conditions. Lane 1: FeFe protein isolated from DJ2560 (*anfO*$^{+}$ *nifEN*$^{+}$); lane 2: FeFe protein isolated from DJ2821 (Δ*anfO nifEN*$^{+}$); lane 3: FeFe protein isolated from DJ2821 (Δ*anfO nifEN*$^{+}$) grown in the presence of 1 μM Mo; lane 4: FeFe protein isolated from DJ2831 (Δ*anfO* Δ*nifEN*) grown in the presence of 1 μM Mo. Refer to Tables S1 and S2 for a complete genotypic description of strains. "MW": standard indicating the molecular weight in kDa. Protein identities are indicated by the arrows. Note that the δ subunit is associated with the FeFe protein α- and β-subunits in all the samples analyzed. AccA is the biotin-binding subunit of acetate carboxylase which is unrelated to the FeFe protein but is also isolated from *A. vinelandii* crude extracts using the Strep-Tactin based affinity purification method. The catalytic properties of these isolated FeFe proteins are shown in Table 1.

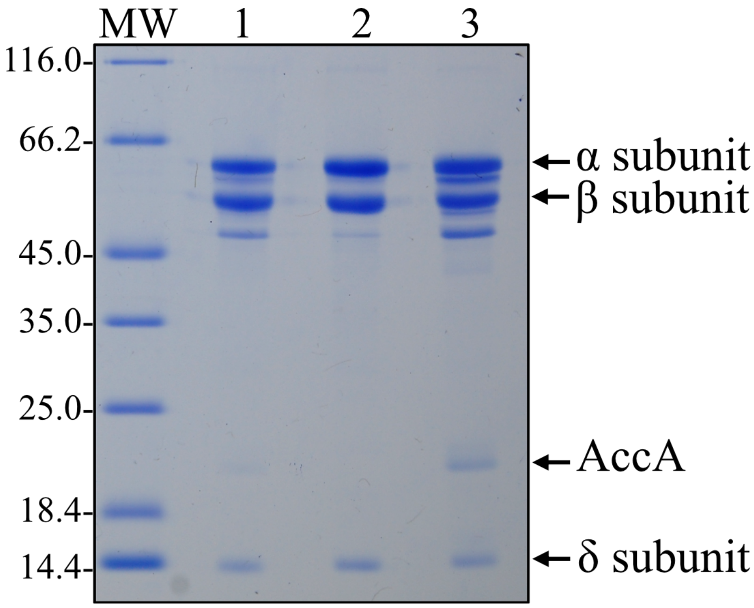

Figure S2. Coomassie blue stained SDS-PAGE of FeFe proteins containing FeFe-, FeV-, or FeMo- cofactor. Lane 1: FeFe protein containing FeFe-cofactor from DJ2241 (*anfO*$^+$) grown in the absence of Mo and V; lane 2: FeFe protein containing FeV-cofactor from DJ2290 (Δ*anfO vnfEN*$^+$) grown in the presence of 5μM V; lane 3: FeFe protein contain FeMo-cofactor from DJ2821 (Δ*anfO nifEN*$^+$) grown in the presence of 1 μM Mo. Refer to Tables S1 and S2 for a complete genotypic description of strains. "MW": standard indicating the molecular weight in kDa. Protein identities are indicated by the arrows. Note the δ-subunit is associated with the FeFe protein αβ-subunits in all the samples analyzed.

A

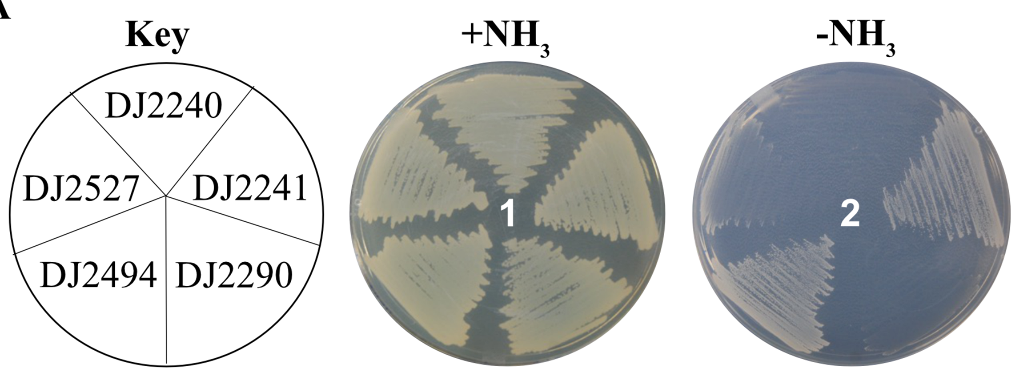

B

Strain	Relevant genotype
DJ2240	Δ*nifDK* Δ*vnfDGK* Δ*anfDGK*
DJ2241	Δ*nifDK* Δ*vnfDGK* *anfD*$^{\text{S-TAG}}$
DJ2290	Δ*nifDK* Δ*vnfDGK* *anfD*$^{\text{S-TAG}}$ Δ*anfO*
DJ2494	Δ*nifDK* Δ*vnfDGK* *anfO*$^{\text{S-TAG (C-terminal)}}$
DJ2527	Δ*nifDK* Δ*vnfDGK* *anfO*$^{\text{S-TAG (N-terminal)}}$

Figure S3. Phenotypic characterization of *Azotobacter vinelandii* strains containing a Strep-tag at the N- or C- terminus of AnfO. A) Strains requiring Fe-only nitrogenase for diazotrophic growth were cultured on Burk's medium agar plates containing a fixed nitrogen source (plate 1) or under diazotrophic conditions (plate 2). Strains were cultured at 30 ºC for 5 days. B) Relevant genes inactivated for each strain. A complete genotypic description of each strain is indicated in Tables S1 and S2. The key observation is that a Strep-tag ("S-TAG") placed at the C-terminus of AnfO (DJ2494) does not impair the *in vivo* function of the protein, while placement of a Strep-tag at the N-terminus of AnfO (DJ2527) or the deletion of a C-terminal portion of AnfO (DJ2290, also DJ2821 shown in Fig. 3) inactivates the *in vivo* function of AnfO based on the diazotrophic growth phenotypes of the corresponding strains.

A

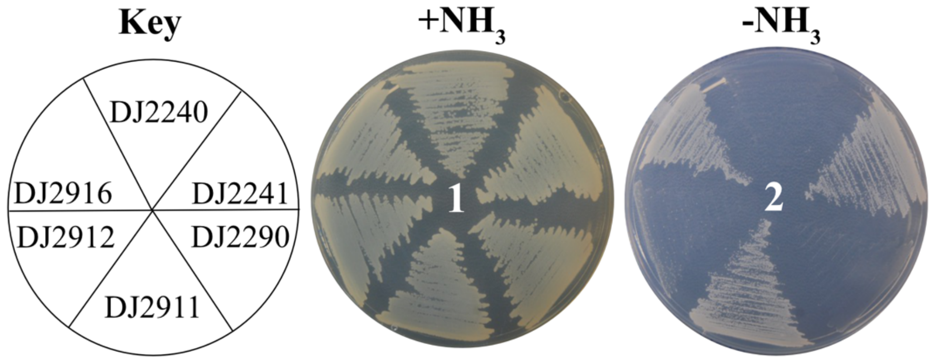

B

Strain	Relevant genes inactivated
DJ2240	*nifDK, vnfDGK, anfDGK*
DJ2241	*nifDK, vnfDGK*
DJ2290	*nifDK, vnfDGK, anfO*
DJ2911	*nifDK, vnfDGK, anfO*^{C159A}
DJ2912	*nifDK, vnfDGK, anfO*^{H203L}
DJ2916	*nifDK, vnfDGK, anfO*^{C201A}

Figure S4. Phenotypic characterization of *Azotobacter vinelandii* strains containing amino acid substitutions within AnfO. A) Strains requiring Fe-only nitrogenase for diazotrophic growth were cultured on Burk's medium agar plates containing a fixed nitrogen source (plate 1) or under diazotrophic conditions (plate 2). Strains were cultured at 30 ºC for 5 days. B) Relevant genes inactivated for each strain and specific amino acid substitutions of AnfO are indicated. A complete genotypic description of each strain is indicated in Tables S1 and S2. AnfO produced by DJ2911 has the conserved Cys^{195} residue substituted by Ala, DJ2912 has the conserved AnfO His^{203} residue substitute by Leu, and DJ2916 has the conserved AnfO Cys^{201} residue substituted by Ala.

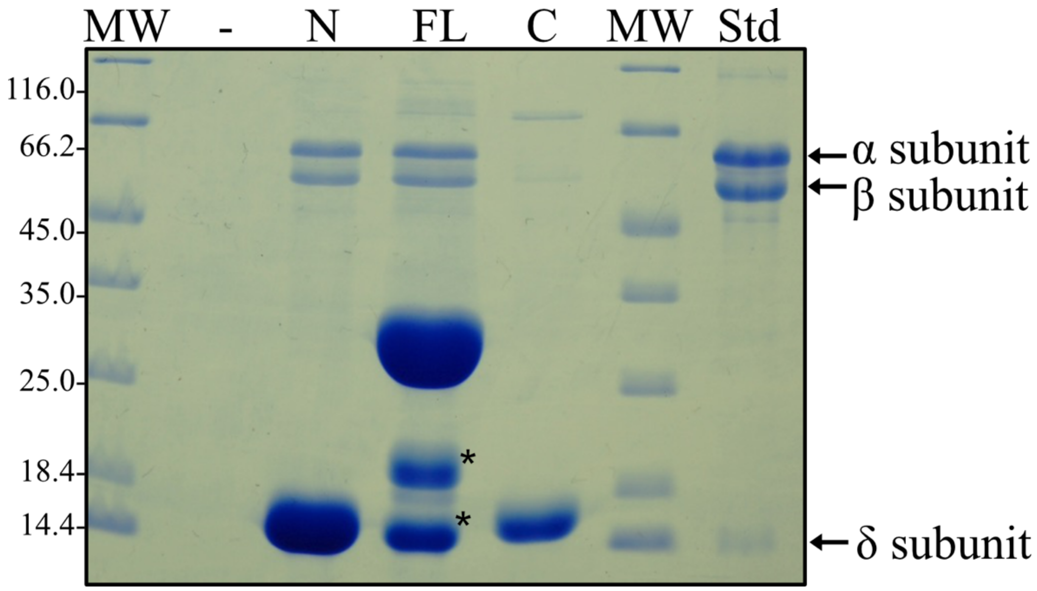

Figure S5. The apo-FeFe protein interacts with the N-terminal domain of AnfO. Strep-tagged versions of purified full-length AnfO ("FL"), its N-terminal domain ("N"), or its C-terminal domain ("C") produced in *E. coli* were used to saturate individual Strep-Tactin columns and washed with buffer B as described in Experimental Procedures. Cell extract of DJ2520 (Δ*nifB*, Δ*nifDK*, and Δ*vnfDGK*) was then passed over each column and washed with buffer B containing no biotin, followed by elution with buffer B containing 50 mM biotin. Because DJ2520 carries a *nifB* deletion the FeFe protein, it does not produce FeFe-cofactor and does not contain the δ-subunit. The Coomassie blue stained SDS-PAGE analysis of the eluted samples is shown. Full-length AnfO and its N-domain capture the apo-FeFe protein α- and β-subunits, whereas the AnfO C-domain did not. The second lane "-" represents a negative control for which no bait protein was immobilized on the Strep-Tactin column. MW = molecular weight standards (kDa), FL = immobilized full-length AnfO used as bait, N = immobilized AnfO N-domain used as bait, C = immobilized AnfO C-domain used as bait, Std = purified FeFe protein standard with the αβδ subunits indicated by arrows. The two smaller proteins indicated by asterisks in the lane labelled "FL" are full-length AnfO cleavage products. The identity of all proteins was confirmed by Mass Spectrometry.

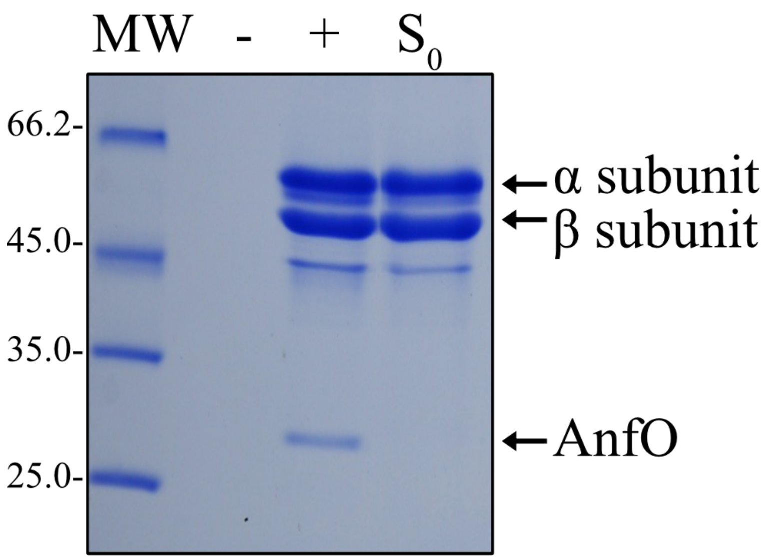

Figure S6. Immobilized apo-FeFe protein interacts with AnfO. Purified Strep-tagged apo-FeFe protein produced by *A. vinelandii* strain DJ2245 was used to saturate individual Strep-Tactin columns and washed with buffer B as described in Experimental Procedures. Cell extract of *E. coli* BL21 cells expressing a plasmid containing a non-tagged version of full-length AnfO (pDB2343) was then passed over each column and washed with buffer B containing no biotin, followed by elution with buffer B containing 50 mM biotin. Coomassie blue stained SDS-PAGE analysis of the eluted samples is shown. The apo-FeFe protein α- and β-subunits capture full-length AnfO as shown in lane "+". Lane "S_0" shows an immobilized apo-FeFe protein sample for which no *E. coli* cell extract was passed over the column. Lane "-" represents a negative control for which no bait protein was immobilized on the Strep-Tactin column. MW = molecular weight standards (kDa). Protein identities are indicated by arrows. Identity of full-length AnfO was confirmed by Mass Spectrometry.
