# Supplementary material for: Molecular sorting of nitrogenase catalytic cofactors
Source: bioRxiv. 2025 Jan 21:2025.01.21.634024. Preprint. [Version 1] doi: 10.1101/2025.01.21.634024 (PMC11785038; doi:10.1101/2025.01.21.634024)
Supplement: Supplement 2 [file media-2.pdf]

Table S1. List of *Azotobacter vinelandii* strains.

Strain	Genotype
DJ2239	*ΔnifDK*, *vnfDGK*::sm^R,rif^R, Δ42kb[a]
DJ2240	*ΔnifDK*, *vnfDGK*::sm^R, *anfDGK*::km^R, rif^R, Δ42kb[a]
DJ2241	*ΔnifDK*, *vnfDGK*::sm^R, $anfD^{S\text{-}TAG}$, rif^R, Δ42kb[a]
DJ2245	*ΔnifDK*, *vnfDGK*::sm^R, Δ*nifB*::km^R, $anfD^{S\text{-}TAG}$, rif^R, Δ42kb[a]
DJ2290	*ΔnifDK*, *vnfDGK*::sm^R, Δ*anfO*, $anfD^{S\text{-}TAG}$, rif^R, tet^R, Δ42kb[a]
DJ2494	*ΔnifDK*, *vnfDGK*::sm^R, $anfO^{S\text{-}TAG\ \text{(C-term)}}$, rif^R, tet^R, Δ42kb[a]
DJ2520	*ΔnifDK*, *vnfDGK*::sm^R, Δ*nifB*, rif^R, Δ42kb[a]
DJ2527	*ΔnifDK*, *vnfDGK*::sm^R, $anfO^{S\text{-}TAG\ \text{(N-term)}}$, rif^R, tet^R, Δ42kb[a]
DJ2560	*ΔnifDK*, *vnfDGK*::sm^R, *vnfE*::gm^R, Δ*modE1*[a], $anfD^{S\text{-}TAG}$, rif^R
DJ2821	*ΔnifDK*, *vnfDGK*::sm^R, *vnfE*::gm^R, Δ*modE1*[a], Δ*anfO*, $anfD^{S\text{-}TAG}$, rif^R
DJ2831	*ΔnifDK*, *vnfDGK*::sm^R, *vnfE*::gm^R, Δ*modE1*[a], Δ*anfO*, *nifE*::km^R, $anfD^{S\text{-}TAG}$, rif^R
DJ2911	*ΔnifDK*, *vnfDGK*::sm^R, $anfD^{S\text{-}TAG}$, $anfO^{C159A}$, Δ42kb[a]
DJ2912	*ΔnifDK*, *vnfDGK*::sm^R, $anfD^{S\text{-}TAG}$, $anfO^{H203L}$, Δ42kb[a]
DJ2916	*ΔnifDK*, *vnfDGK*::sm^R, $anfD^{S\text{-}TAG}$, $anfO^{C201A}$, Δ42kb[a]

$anfD^{S\text{-}TAG}$: Strep-tag is placed at the C-terminal of *anfD;* rif^R: rifampicin; sm^R: streptomycin; km^R: kanamycin; tet^R: tetracycline; gm^R: gentamycin.
Location of residues removed and/or placement of insertions are indicated in Table S2.
[a] W-tolerance is the result of a Δ42kbp in a genomic deletion required for Mo acquisition or the Δ*modE1* whose product is involved in regulating Mo acquisition and Mo-dependent repression of *anf* gene expression.

Table S2. List of plasmids used for the construction of *Azotobacter vinelandii* strains.
Location of residues removed and/or placement of insertions are indicated. Nomenclature corresponds to the genotype shown in Supplemental Table 1. S-TAG: Strep-tag (ASWSHPQFEK); km^R: kanamycin; sm^R: streptomycin; gm^R: gentamycin.

**Plasmid**	**Deletion/Insertion**	**Residues Removed/ Insertion Location**
pDB33	Δ*nifDK*	NifD^{103} - NifK^{308}
pDB161	Δ*nifB*	NifB$^{60\text{-}307}$
pDB218	*nifB*::km^R	NifB$^{60\text{-}307}$
pDB259	*nifE*::km^R	NifE$^{15\text{-}261}$
pDB2134	*anfDGK*::km^R:	AnfD^{204} - AnfK^{148}
pDB2139	*vnfDGK*::sm^R	VnfD^{271} - VnfK^{202}
pDB2158	*anfD*$^{S\text{-}TAG}$	AnfD^{518}
pDB2200	*vnfE*::gm^R	VnfE^{91}
pDB2224	Δ*anfO*	AnfO$^{179\text{-}224}$
pDB2265	Δ*modE1*	ModE1$^{151\text{-}215}$
pDB2355	*anfO*$^{S\text{-}TAG\ (C\text{-}term)}$	AnfO^{245}
pDB2395	*anfO*$^{S\text{-}TAG\ (N\text{-}term)}$	AnfO^{1}
pDB2611		AnfO^{C159A}
pDB2612		AnfO^{C201A}
pDB2613		AnfO^{H203L}

Table S3. Plasmids used for heterologous expression of AnfO and the N- and C-domains in *Escherichia coli* BL21(DE3) competent cells.

Plasmid	Description
pDB2343	For purification of the non-tagged, full-length AnfO (residues 1-245).
pDB2418	For purification of the full-length AnfO (residues 1-245). A Strep-tag (ASWSHPQFEK) is located after residue 245.
pDB2526	For purification of the C-terminal domain of AnfO (residues 138-245). A TwinStrep-tag (ASWSHPQFEKGGGSGGGSGGSAWSHPQFEKAS) is located before residue 138.
pDB2554	For purification of the N-terminal domain of AnfO (residues 1-132). A Strep-tag (ASWSHPQFEK) is located after residue 132.

Table S4. Quantification of FeMo-cofactor binding to AnfO based ICP-MS metal and BCA protein assays.

Sample	Fe	Mo	AnfO	[Mo] : [AnfO]
1	8.4	1	1.6	0.62
2	7.1	1	2.3	0.44
3	6.6	1	1.9	0.53
4	11	1	2.2	0.45
**Combined**	**7.1**	**1**	**2.0**	**0.51**

The "Combined" sample was prepared by mixing samples 1-4 and concentration using a stirred cell concentrator. These data show that repeated generation of AnfO containing FeMo-cofactor results in an [Fe] : [Mo] ratio close to 7:1, as is expected for a FeMo-cofactor containing protein. Some occurrences of higher [Fe] : [Mo] ratio have been observed (notably sample 4), which we attribute to cluster degradation. Additionally, the [Mo] : [AnfO] ratio, which we use as a proxy for the [FeMo-cofactor] : [AnfO] ratio, consistently resides in the range of 0.5 ± 0.1. At this time, we cannot distinguish between weak FeMo-cofactor binding to AnfO (and a native 1:1 cofactor:AnfO ratio) or a native 1:2 cofactor:AnfO ratio.
